# A Systematic Review of Case Reports on the Neck-Tongue Syndrome

**DOI:** 10.3390/medicina57101097

**Published:** 2021-10-13

**Authors:** Hyunjoong Kim, Seungwon Lee, Namjeong Cho, Seonghyeok Song

**Affiliations:** 1 Manual Therapy Center, Chung Ha Lin Hospital, 61 Gaegeum-gil, Gwangju 62070, Korea; hyun-joongkim@nmslab.org; 2 Department of Physical Therapy, Sahmyook University, 815 Hwarang-ro, Seoul 01795, Korea; swlee@syu.ac.kr; 3 Department of Physical Therapy, Kyungbuk College, 77 Daehak-ro, Yeongju 36133, Korea; 12mjnj@naver.com; 4 Ez Rehabilitation Medical Center, 302 Gwanggyojungang-ro, Yongin 16943, Korea

**Keywords:** headache, manual therapy, neck pain, neck-tongue syndrome, posterior neck pain

## Abstract

*Background and Objectives*: Neck-tongue syndrome (NTS) is rare, and characterized by unilateral upper neck or occipital pain and paresthesia in the ipsilateral hemisphere of the tongue due to neck movement. Treatment for NTS is mainly conservative, but the symptoms, causes, and rationale for treatment remain controversial. This study aimed to provide a framework for NTS treatment in clinical practice based on recent treatment directions. *Materials and Methods*: Case reports published from the past 20 years to August 2021 were searched through MEDLINE, EMBASE, and PEDro databases. Since there is no established management for NTS, the search terms were neck-tongue syndrome and case reports. The Critical Appraisal Checklist for Case Reports was used for the quality assessment of case reports. Through descriptive analysis, NTS symptoms, interventions, and results were reviewed. *Results*: Among the 16 studies searched, six case reports were selected and analyzed based on eight criteria. Symptoms included neck pain and ipsilateral tongue paralysis when the head was turned. As an intervention, six and four studies showed immediate symptom relief through manual therapy and exercise, respectively. *Conclusions*: Based on the reviewed evidence, management through physical therapy and chiropractic therapy with conservative methods such as manual therapy and exercise for patients with neck-tongue syndrome is recommended.

## 1. Introduction

Neck-tongue syndrome (NTS) is a very rare clinical condition defined by the International Headache Society (IHS) as “painful lesions of the cranial nerves and other facial pain” [1]. Characteristic symptoms of NTS include unilateral upper neck or occipital pain, and paresthesia of the ipsilateral half of the tongue due to neck movement [2]. Past studies of prevalence have shown that NTS is a rare case, estimated to occur in 0.22% of adults [3].

According to the reported characteristics of NTS, it can be divided into uncomplicated NTS and complicated NTS [4]. Uncomplicated NTS is related to idiopathic, hereditary, and trauma, and complicated NTS is reported to appear in patients with underlying diseases [5]. For this reason, differential diagnosis is considered very important, and related diseases include cervical artery dysfunction, vertebrobasilar insufficiency (VBI), transient ischemic attack, migraine, ligamentous insufficiency, and Chiari-1 malformation [2,6,7,8].

In the study of Lewis et al. [9], an autosomal dominant genetic pattern was reported through three cases as a genetic trait, and although symptoms were present in adolescence, the symptoms disappeared in adulthood. For this reason, it can be suggested that the pathogenesis according to age may be different. In other words, structural abnormalities in NTS appearing in adults are found, but rarely reported in adolescents. The pathogenesis reported so far is that stimulation of the C2 ventral branch, which is transmitted through the afferent proprioceptive nerve from the lingual nerve to the C2 nerve root and cervical plexus through anastomosis with the sublingual nerve, causes tongue paresthesia [5,10].

Classification of reported treatments for NTS can be divided into pharmacological and non-pharmacological treatments. Pharmacological treatments include antiepileptic drugs, antidepressants, neuropathic pain modulators, and nonsteroidal anti-inflammatory drugs [7,11,12]. Non-pharmacological treatments include cervical collar, physical therapy, exercise, and neuromuscular re-education [4,11,13].

In fact, the evidence for the treatment of NTS is limited, and its symptoms and causes are controversial. Therefore, the purpose of this study is to provide a framework for NTS treatment in clinical practice based on the latest treatment directions and case analysis in case reports published over the past 20 years.

## 2. Materials and Methods

This is a systematic review that integrates and analyzes case reports for NTS. This study followed The Preferred Reporting Items for Systematic Reviews and Meta-Analysis (PRISMA) guidelines, and the study protocol was registered in the International Prospective Register of Systematic Reviews (PROSPERO) before the study was conducted (registration number: CRD42021245556).

### 2.1. Data Sources and Searches

International databases such as MEDLINE (Ovid), EMBASE, and PEDro were used to search the literature. The search terms were a combination of the keyword (neck-tongue syndrome) and types of studies (case reports and case series). Studies within the past 20 years are included in order to include relatively recent studies in the review. The literature was searched using only the above-mentioned databases, and the last literature search was conducted in August 2021. Searches were performed by two researchers (Kim, H. and Song, S.).

### 2.2. Study Selection and Data Extraction

First, articles searched through these databases were duplicated using a bibliographic citation management software (EndNote X9, Thomson Reuters, NY, USA). The original title and abstract texts of the papers were reviewed. The general characteristics of the studies, characteristics of the intervention, and study results were extracted. Two researchers independently searched the data, as well as selected and extracted them. If the independently extracted data from both researchers did not match, the original text was reviewed to reach a consensus.

### 2.3. Evaluating the Risk of Bias

The Critical Appraisal Checklist for Case Reports developed by Moola et al. [14] was used to perform a quality check on the systematic review of case reports. If five out of the eight evaluation criteria were met, the quality was judged sufficient. All the researchers agreed on the searched studies.

### 2.4. Data Analyses

The nature of case reports, such as the heterogeneity of study design, the health status of the participants, specific intervention and testing procedures, and outcome variables, made meta-analysis impossible. Therefore, descriptive analysis was performed.

## 3. Results

### 3.1. Literature Search

**Figure 1:**
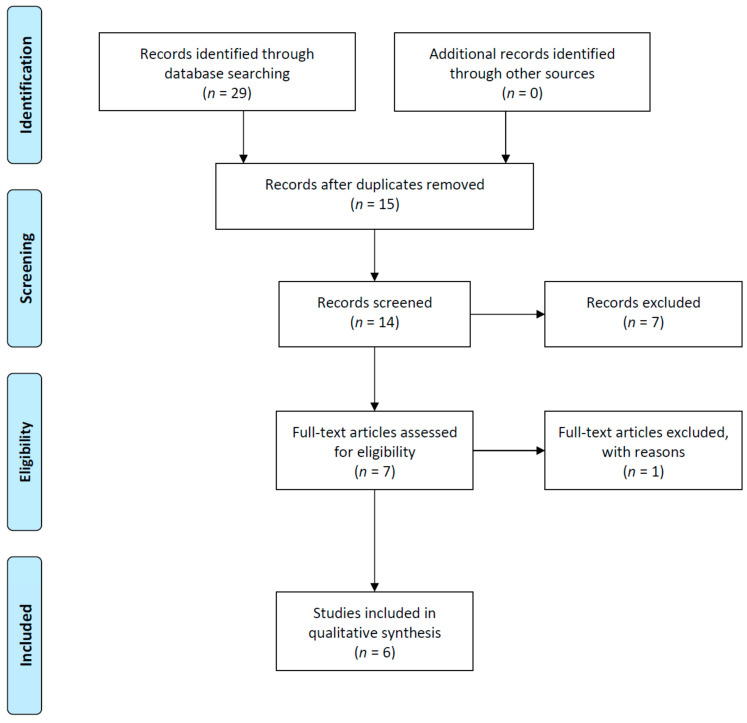
PRISMA flow diagram.

From the search results of all databases, 16 studies were identified and subsequently reviewed. After de-duplication, 14 studies were selected, and seven studies were excluded through abstract review. Furthermore, six non-English studies were excluded from the original text review. Finally, six cases of NTS reported in the last 20 years were included in the analysis. The PRISMA flow diagram for the systematic review is shown in Figure 1.

### 3.2. Evaluating the Risk of Biases

Table 1 shows the risk of bias evaluated in this study using the Critical Appraisal Checklist for Case Reports. The first evaluation criterion was patient demographics; all six studies described sex, age, and sometimes occupation (Yes: 6). The second was patient histories and timelines; only one out of six studies clearly described these (Yes: 1, No: 3, Unclear: 2). The third criterion was a proper description of the current clinical condition of the patients; all six studies described this properly (Yes: 6). The fourth criterion was a clear description of the clinical condition, evaluation, and results; all six studies described these in detail (Yes: 6). The fifth evaluation criterion is a clear description of intervention and treatment procedures; general statements were made regarding these in all six studies, but specific details on treatment procedures or intervention intensity were insufficient (Yes: 3, No: 2, Unclear: 1). The sixth criterion was a clear description of post-intervention clinical conditions; these were not described in as much detail as pre-intervention conditions, but pain and symptom relief were clearly described in most of the six studies (Yes: 4, No: 2). The seventh criterion was information on adverse or unanticipated events; these do not apply to NTS, since mainly conservative treatment was performed. Finally, the eighth criterion was if the case report provided takeaway lessons; the six studies were judged to be useful because various tests and detailed explanations were described for rare cases in these studies.

In the evaluated case reports, the demographic characteristics of NTS patients, the patient’s medical history according to the timeline, the current clinical status of patients, the diagnostic test or evaluation method, and the results were all reported appropriately.

### 3.3. Cases of Neck-Tongue Syndrome

Table 2 summarizes the characteristic symptoms of the neck and tongue for NTS and the results of the interventions and treatments. In the six case reports [4,13,15,16,17,18], symptoms and treatments were relatively similar. Although patients’ ages were similar, most patients were in their 20s or younger, and the number of women was relatively high.

The symptoms mainly occurred during head-turning, and were characterized by pain from the neck to the upper neck or occiput and ipsilateral tongue numbness. In addition, visits to the clinic occurred after some months. Most of the interventions for NTS were manual therapy and exercise with non-pharmacological treatments, and in some cases, symptoms were relieved with pharmacological treatments. Symptoms improved greatly in the short term. However, rather than being completely cured, some symptoms remained even after some years, though at a considerably lesser intensity.

## 4. Discussion

This systematic review was performed to analyze case reports on NTS. Of the 16 studies searched in the databases, only six were selected and analyzed. The results of the quality assessment showed that all six studies were sufficient.

In the analyzed case reports, as defined in the study of Lance and Anthony [2], there were unilateral upper neck or occipital pain and paresthesia in the ipsilateral hemisphere of the tongue due to neck movement. This caused neck pain due to the sudden rotation. In addition, accompanying symptoms not reviewed in this study included dysarthria, numbness of the mastoid process region, facial paresthesia, and numbness in the upper extremities [2,7,9]. As a further note, in a systematic review reported in 2018, 39 patients were analyzed [19]. In this study, most of these patients had NTS onset at childhood or adolescence, and those affected were mostly women (56%). Ligament laxity during growth and development and some genetic factors were reported to be causes of the early onset age. In other studies on NTS episodes, there were cases of cervical osteoarthritis [3,7,20], and in magnetic resonance imaging (MRI) studies, there were cases of retrocerebellar arachnoid cysts [21].

Although non-surgical methods for managing NTS are common, a study on the surgical management of NTS confirmed that symptoms were relieved by resolving the compression on the C2 spinal nerve by the protuberant atlantoaxial joint [22]. In the pharmacological and non-pharmacological interventions included in non-surgical interventions, pharmacological interventions were performed only in the Allen et al. [18] study. In the other five case reports, manual therapy, exercise, and cervical collar were found to be effective.

Differential diagnoses of idiopathic and post-traumatic NTS can be made by considering the accompanying symptoms. Treatment may include using a neck collar or medication to relieve symptoms, and manual therapy and exercise appear to be appropriate as an intervention. However, in a review by Gelfand et al. [19], pathologically, when ankylosing spondylitis and rheumatoid arthritis are accompanied by chronic pain and joint instability in the cervical spine (C1, C2), it is difficult to explain the pain of NTS neurologically. Furthermore, what can be said to be different from our study is that there is no advantage of spinal manipulation therapy for the type of suspected instability. However, in childhood and adolescence cases, there are cases of compression due to shortness of the scalenus and congenital abnormalities or shortness of the sternocleidomastoid muscle. At this time, symptoms are improved through the effect of manual therapy for the absence of neuromuscular control development of muscles and ligaments due to neurophysiological pathology and structural instability [23]. Therefore, it is suggested that interventions such as spinal manipulation therapy, manual therapy, exercise, and neuromuscular re-education can be helpful as a conservative treatment method.

Although it is a syndrome with a relatively low incidence and long-term discomfort, randomized controlled trials are difficult to conduct, but it is worth studying the most effective management through a long-term cohort study. In addition, in items b, c, and d of ICHD-3, first, diagnosis of intermittent pain rather than chronic pain when there is no neck movement, second, sharp pain during rotation, accompanied by paresthesia of the tongue, and third, there is partial agreement as it is essential for the diagnosis of NTS pain that the duration of treatment ranges from a few seconds to a few minutes.

## 5. Conclusions

This systematic review was performed to synthesize and analyze case reports on NTS. Although there are not many cases in the world, we confirmed some agreements in these reports as regards to the diagnosis and treatment of NTS. Although it is difficult to establish clear treatment recommendations, management through manual therapy and exercise prevailed in a number of cases.

## Figures and Tables

**Table 1:** Critical appraisal checklist for case reports.

Critical Appraisal Checklist	Roberts. [15]	Niethamer and Myers. [13]	Borody. [16]	Chirchiglia et al. [4]	Chu and Lin. [17]	Allen et al. [18]
1. Were the patient’s demographic characteristics clearly described?	Yes	Yes	Yes	Yes	Yes	Yes
2. Was the patient’s history clearly described and presented as a timeline?	Unclear	Yes	No	No	No	Unclear
3. Was the current clinical condition of the patient on presentation clearly described?	Yes	Yes	Yes	Yes	Yes	Yes
4. Were diagnostic tests or assessment methods and the results clearly described?	Yes	Yes	Yes	Yes	Yes	Yes
5. Was the intervention(s) or treatment procedure(s) clearly described?	Yes	Yes	No	Unclear	Yes	No
6. Was the post-intervention clinical condition clearly described?	Yes	Yes	Yes	No	Yes	Yes
7. Were adverse events (harms) or unanticipated events identified and described?	Unclear	Yes	Unclear	No	Unclear	Yes
8. Does the case report provide takeaway lessons?	Yes	Yes	Yes	Yes	Yes	Yes

**Table 2:** Characteristics of the case reports.

Study	Sex	Age	Symptoms				Interventions		Results	Author’s Conclusion
			Triggers	Neck	Tongue	NTS Duration	Types	Intensity		
Roberts. [15]	Female	34	Rotation of the head to the left side	Mild pain in the left suboccipital region	Described as “tingling,” “shooting,” “piercing,” and “electric shock-like” on the left side tongue	>2 years	Spinal manipulative therapy, myofascial release, and exercises	Two weeks	Symptoms improved after 2 weeks of intervention and persisted after 2 years	Chiropractic care is helpful for NTS
Niethamer and Myers. [13]	Female	13	Turning head to one side or the other or looking up	Sharp pain	Numbness/tingling of the ipsilateral tongue	> 6 months	Manual therapy, exercise (including postural awareness)	Eight visits	Patient-specific functional scale, cervical ROM, postural normalization	An effective known treatment for cervicogenic headache may be the basis for treating NTS
Borody. [16]	Female	24	Brisk active rotation	Upper neck (right) ‘‘sharp’’ or ‘‘piercing’’ pain	Ipsilateral tongue numbness	Recurrent pain after 8 years of age	Diversified spinal manipulative therapy performed to the restrictions throughout the cervical spine	Various intervals	Reduction in the frequency and intensity of the symptoms	In the absence of other additional symptoms and upper cervical instability, spinal manipulative therapy is effective
Chirchiglia et al. [4]	Female	42	Rotation of the head to the right side	Severe neck pain	Paresthesia of the right tongue	A month later, reported a whiplash injury, following a car accident	Topiramate combined with non-steroidal anti-inflammatory drugs and cervical collar	Two weeks	Improved pain both in the neck and the tongue, reducing the episodes in frequency and intensity	Pharmacological treatment is prophylactic
Chu and Lin. [17]	Male	47	Head-turning to the right, forward neck glide or chewing	right suboccipital pain	Tingling of the right face and ipsilateral hemitongue	>3 months	Chiropractic regimen	Three times a week for four weeks	The painful episodes had disappeared and the active range of neck motion had increased	Conservative management, including physiotherapy and minor cervical adjustment, may be the preferred initial treatment
Allen et al. [18]	Female	11	Head-turning	Severe shooting/burning pain in the posterior neck	Ipsilateral hemianesthesia	>5 months	Gabapentin and topical lidocaine	-	Less pain intensity at follow-up after 1 year	It is characterized by additional symptoms of headache; headache was spontaneously resolved in 2 patients, and 1 patient was relieved of headaches by gabapentin
	Female	11	Sudden head-turning/neck movement, fatigue	Severe burning pain in the posterior neck	Ipsilateral numbness	After symptom onset	-	4 years follow-up	Relieve symptoms naturally	
	Male	12	Sudden head-turning	Severe burning pain in the Occiput	Ipsilateral tightness/twisting	Some months		6 years follow-up		

## References

[1] Hu N., Dougherty C. (2016). Neck-tongue syndrome. Curr. Pain Headache Rep..

[2] Lance J.W., Anthony M. (1980). Neck-tongue syndrome on sudden turning of the head. J. Neurol. Neurosurg. Psychiatry.

[3] Sjaastad O., Bakketeig L. (2006). Neck-tongue syndrome and related (?) conditions. Cephalalgia.

[4] Chirchiglia D., Della Torre A., Chirchiglia P., Pugliese D., Gabriele D. (2019). The neck-tongue syndrome following cervical whiplash injury. Interdiscip. Neurosurg..

[5] Bogduk N. (1981). An anatomical basis for the neck-tongue syndrome. J. Neurol. Neurosurg. Psychiatry.

[6] Evans R.W. (2006). Case studies of uncommon headaches. Neurol. Clin..

[7] Fortin C.J., Biller J. (1985). Neck tongue syndrome. Headache.

[8] Wong S., Paviour D., Clifford-Jones R. (2008). Chiari-1 malformation and the neck-tongue syndrome: Cause or coincidence?. Cephalalgia.

[9] Lewis D.W., Frank L.M., Toor S. (2003). Familial neck-tongue syndrome. Headache.

[10] Toure G., Bicchieray L., Selva J., Vacher C. (2005). The intra-lingual course of the nerves of the tongue. Surg. Radiol. Anat..

[11] Chedrawi A.K., Fishman M.A., Miller G. (2000). Neck-tongue syndrome. Pediatr. Neurol..

[12] Queiroz L.P. (2013). Unusual headache syndromes. Headache.

[13] Niethamer L., Myers R. (2016). Manual therapy and exercise for a patient with neck-tongue syndrome: A case report. J. Orthop. Sports Phys. Ther..

[14] Moola S., Munn Z., Tufanaru C., Aromataris E., Sears K., Sfetc R., Currie M., Lisy K., Qureshi R., Mattis P. (2017). Chapter 7: Systematic reviews of etiology and risk. Joanna Briggs Institute Reviewer’s Manual 2017.

[15] Roberts C.S. (2016). Chiropractic management of a patient with neck-tongue syndrome: A case report. J. Chiropr. Med..

[16] Borody C. (2004). Neck-tongue syndrome. J. Manip. Physiol. Ther..

[17] Chu E.C.P., Lin A.F.C. (2018). Neck–tongue syndrome. BMJ Case Rep..

[18] Allen N.M., Dafsari H.S., Wraige E., Jungbluth H. (2018). Neck-tongue syndrome: An underrecognized childhood onset cephalalgia. J. Child. Neurol..

[19] Gelfand A.A., Johnson H., Lenaerts M.E., Litwin J.R., De Mesa C., Bogduk N., Goadsby P.J. (2018). Neck-Tongue syndrome: A systematic review. Cephalalgia.

[20] Queiroz L., Cavallazzi L. (1999). Neck-tongue syndrome with twisting of the tongue: Report of two cases. Cephalalgia.

[21] Evans R.W., Lance J.W. (2000). Expert opinion: Transient headache with numbness of half of the tongue. Headache.

[22] Elisevich K., Stratford J., Bray G., Finlayson M. (1984). Neck tongue syndrome: Operative management. J. Neurol. Neurosurg. Psychiatry.

[23] Song S., Hwang W., Lee S. (2021). Effect of physical therapy intervention on thickness and ratio of the sternocleidomastoid muscle and head rotation angle in infants with congenital muscular torticollis: A randomized clinical trial (CONSORT). Medicine.

